# Resuscitative endovascular balloon occlusion of the aorta performed by emergency physicians for traumatic hemorrhagic shock: a case series from Japanese emergency rooms

**DOI:** 10.1186/s13054-018-2032-y

**Published:** 2018-04-21

**Authors:** Ryota Sato, Akira Kuriyama, Rei Takaesu, Nobuhiro Miyamae, Wataru Iwanaga, Hayato Tokuda, Takehiro Umemura

**Affiliations:** 1 Department of Emergency and Critical Care Medicine, Urasoe General Hospital, 4-16-1, Iso, Urasoe-city, Okinawa 901-2131 Japan; 2 Department of Internal Medicine, John A. Burns School of Medicine, University of Hawaii at Manoa, Honolulu, HI USA; 3 Emergency and Critical Care Center, Kurashiki Central Hospital, Okayama, Japan; 4 Department of Emergency Medicine, Okinawa Prefectural Nannbu Medical Center, Okinawa, Japan; 5 Department of Emergency Medicine, Rakuwakai Otowa Hospital, Kyoto, Japan; 6 Department of Emergency Medicine, Kenwakai Ohtemachi Hospital, Fukuoka, Japan

**Keywords:** Hemorrhagic shock, Severe trauma, Resuscitative endovascular balloon occlusion of the aorta, REBOA

## Abstract

**Background:**

Resuscitative endovascular balloon occlusion of the aorta (REBOA), which has been increasingly used for the management of hemorrhagic shock, is a less invasive strategy for the management of patients with very severe hemorrhage. However, its effectiveness remains controversial.

**Methods:**

This retrospective case series included trauma patients who underwent REBOA for hemorrhagic shock due to trauma in four Japanese tertiary care emergency centers from January 2013 to March 2017. Patients in cardiac arrest at the time of REBOA and those who underwent REBOA for nontraumatic causes during the study period were excluded.

**Results:**

A total of 24 patients underwent REBOA during the study period. The median age was 52 years (interquartile range (IQR) 36.5–62.5), 17 (70.8%) of the patients were male, and 23 (95.8%) had blunt trauma. The 24-h survival was 50% (*n* = 12), and the in-hospital survival rate was 41.7% (10/24). In all cases, REBOA was performed in emergency rooms by emergency physicians without fluoroscopic guidance. Complications of REBOA were mesenteric ischemia (*n* = 1, 4.2%), ischemia of the lower extremities (*n* = 1, 4.2%), and placement of REBOA in thoracic aortic injury (*n* = 3, 12.5%).

**Conclusions:**

REBOA can be an effective and feasible tool for controlling massive hemorrhage due to trauma. However, caution should be exercised regarding complications including placement of REBOA in aortic injury and limb ischemia in cases where REBOA is performed in an emergency department setting with minimal or no support from trauma surgeons.

## Background

Hemorrhage is the main cause of preventable death in trauma patients [1–3], with noncompressible torso hemorrhage the most common source in the majority of cases [4]. These patients are at high risk for progression to cardiovascular collapse if the hemorrhage cannot be controlled rapidly. Therefore, several techniques and strategies have been developed to mitigate hemorrhage in trauma.

Resuscitative endovascular balloon occlusion of the aorta (REBOA), which has been increasingly used for the management of hemorrhagic shock, is a less invasive alternative to resuscitative thoracotomy for the management of patients with very severe hemorrhage [5–7]. REBOA restores perfusion by reducing the volume distribution, thereby facilitating an increase in myocardial and cerebral perfusion. In contrast, a previous study showed an association between the use of REBOA and higher mortality in hemodynamically unstable patients with torso trauma [8]. A systematic review also revealed that REBOA did not improve mortality in patients with hemorrhagic shock, albeit there was a successful elevation in blood pressure [9]. However, these studies included trauma surgery care centers with readily available trauma surgeons and interventional radiologists for 24 h a day, 7 days a week. Since severe trauma is uncommon in Japan not many trauma centers exist, and immediate transportation of patients to operating rooms or interventional radiology laboratories might be more beneficial than REBOA. However, since most Japanese tertiary care centers are not specialized in trauma surgery care, we aimed to evaluate the clinical use of REBOA in four standard Japanese emergency rooms that do not specialize in trauma surgery.

## Methods

This retrospective case series included trauma patients who underwent REBOA for hemorrhagic shock due to trauma in four Japanese tertiary care emergency centers, including Urasoe General Hospital, Rakuwakai Otowa Hospital, Okinawa Prefectural Nannbu Medical Center, and Kenwakai Ohtemachi Hospital from January 2013 to March 2017. Patients in cardiac arrest at the time of REBOA and those who underwent REBOA for nontraumatic causes during the study period were excluded. REBOA was considered as part of routine clinical care, and electronic medical charts were reviewed in a retrospective manner. Demographic data, mechanism of injury, Revised Trauma Score (RTS) [10], Injury Severity Score (ISS) [11], and Trauma and Injury Severity Score (TRISS) [12] were obtained and calculated from the electronic charts. Because of the nature of a retrospective study, there were no clearly set criteria to indicate REBOA in the present study. The decision to implement REBOA was at the clinical discretion of the attending emergency physician in charge of each patient. However, systolic blood pressure before implementing REBOA was approximately 80 mmHg in all patients except for two patients whose initial systolic blood pressures were 60 mmHg and 70 mmHg. Their blood pressure temporally elevated to 120 mmHg with fluid resuscitation just before the placement of REBOA. However, their blood pressure fluctuated, and these patients were considered to be nonresponsive to the initial fluid. Therefore, all REBOA were implemented in traumatic patients with shock unresponsive to initial fluid resuscitation. REBOA was achieved using Block Balloon™ (Senko Medical Instrument, Tokyo, Japan) or Rescue Balloon™ (Tokai Medical Products, Aichi, Japan), which were deployed via 10- and 7-French sheaths placed in the femoral artery. This study was approved by the Urasoe General Hospital institutional ethics committee, Okinawa Prefectural Nannbu Medical Center institutional ethics committee, Rakuwakai Otowa Hospital institutional ethics committee, and Kenwakai Ohtemachi Hospital institutional ethics committee.

## Results

A total of 24 patients underwent REBOA during the study period. The median age was 52 years (interquartile range (IQR) 36.5–62.5), 17 (70.8%) of the patients were male, and 23 (95.8%) had blunt traumas. The mechanisms of injury included motor vehicle accidents (*n* = 13, 54.2%), falls (*n* = 8, 33.3%), other blunt trauma (*n* = 2, 8.3%), and stab wounds (n = 1, 4.2%). The median ISS, RTS, and TRISS were 35 (IQR 26–42), 4.24 (IQR 2.61–6.11), and 0.67 (IQR 0.44–0.95), respectively. The 24-h survival rate was 50% (*n* = 12), and both in-hospital and 30-day survival rates were 41.7% (10/24), as shown in Tables 1 and 2.

**Table 1:** Characteristics of patients who underwent REBOA

Overall *n* = 24	
Age (years)	51.5 (36.5–62.5)
Male, *n* (%)	17 (70.8%)
Blunt injury, *n* (%)	23 (95.8%)
Mechanism of injury, *n* (%)	
Motor vehicle accident	13 (54.2%)
Fall	8 (33.3%)
Other blunt trauma	2 (8.3%)
Stab wound	1 (4.2%)
RTS	4.24 (2.61–6.11)
ISS	35.0 (25.8–42.3)
TRISS	0.67 (0.44–0.95)
24-h survival, *n* (%)	12 (50%)
In-hospital mortality, *n* (%)	7 (29.2%)
30-day mortality, *n* (%)	7(29.2%)

For age, RTS, ISS, and TRISS, medians and interquartile ranges (25th–75th percentile) are shown

*ISS* Injury Severity Score, *REBOA* resuscitative endovascular balloon occlusion of the aorta, *RTS* Revised Trauma Score, *TRISS* Trauma and Injury Severity Score

**Table 2:** Patients included in this study

Age	Sex	Trauma site	Outcome at discharge
65	M	Liver injury	Died
38	M	Liver injury, hemothorax	Died
71	M	Cardiac injury	Died
32	F	Pelvic fracture, lung contusion, TBI	Survived
53	M	Pelvic fracture	Died
40	M	Thoracic aortic injury	Died
56	M	Mesenteric injury	Survived
20	M	Liver injury, kidney injury	Survived
17	M	Hemothorax, retroperitoneal hemorrhage	Died
62	M	Pelvic fracture	Died
74	M	Pelvic fracture	Died
66	M	Pelvic fracture, TBI	Died
86	M	Pelvic fracture	Survived
56	F	Liver injury	Died
45	M	Pelvic fracture	Died
64	F	Thoracic aortic injury	Died
22	M	Lung contusion, TBI	Died
30	M	Pelvic fracture	Survived
47	M	Mesenteric injury	Survived
50	F	Abdominal aortic injury, mesenteric injury	Survived
58	F	Pelvic injury	Survived
39	M	Liver injury, splenic injury	Survived
18	F	Liver injury, kidney injury	Survived
56	F	Liver injury, pelvic fracture	Died

*F* female, *M* male, *TBI* traumatic brain injury

REBOA was performed in emergency rooms by board-certified emergency physicians or fellows under supervision of board-certified emergency physicians in 22 cases and by board-certified surgeons in two cases without fluoroscopic guidance. REBOA was placed blindly without ultrasound in all cases. The placing of the balloon was confirmed with x-ray or fluoroscopy in cases in which transcatheter arterial embolization was performed or computed tomography (CT) scan of the trunk in cases in which REBOA was performed before taking a CT scan. The balloon was placed in zone I in all cases and the balloon was inflated to a diameter between 22.5 mm and 27.5 mm for the management of hypotension. The aorta is usually divided into three zones: zone I is the descending thoracic aorta between the origin of the left subclavian and celiac arteries; zone II is the paravisceral aorta between the celiac and the lowest renal artery; and zone III is the infrarenal abdominal aorta between the lowest renal artery and the aortic bifurcations [13]. The median total occlusion time in patients who tolerated balloon deflation was 65.0 min (IQR 54.8 to 75). Systolic blood pressure immediately after REBOA was significantly higher than that immediately before REBOA (median 70 mmHg versus 98 mmHg; *p* = 0.007). The median time between arrival and balloon inflation (door-to-inflation time) was 56.0 min (IQR 35.5 to 79.8). The causes of death were multiorgan failure (*n* = 8, 33.3%), severe brain injury (*n* = 2, 8.3%), and uncontrollable hemorrhage (*n* = 4, 16.7%). In 21 (87.5%) and 3 (12.5%) cases, 10- and 7-French introducers were used, respectively. After the removal of the introducers manual compression was performed for approximately 15 to 30 min. Subsequently, a compression bandage was placed on the insertion site for 6–8 h. No thrombus was found on the introducers that were removed.

Complications of REBOA included mesenteric ischemia (*n* = 1, 4.2%), ischemia of the lower extremities (*n* = 1, 4.2%), and placement of REBOA in thoracic aortic injury (*n* = 3, 12.5%), as shown in Table 3. All these complications were observed in patients for whom the 10-French introducer was used. Placement of REBOA in thoracic aortic injury was identified by contrast-enhanced CT scan. In these cases, chest x-ray failed to reveal any evidence of thoracic aortic injury. Aortic injuries are classified to four categories: grade I (intimal tear), grade II (intramural hematoma), grade III (pseudoaneurysm), and grade IV (rupture) [14]. Two of three thoracic aortic injuries were rated as grade IV and they died of uncontrollable hemorrhage. In one patient with thoracic aortic injury, REBOA was placed distal to the injury site and aortic injury due to REBOA placement cannot be ruled out. In the other patient, REBOA was placed proximal to the injury site and it was less likely that REBOA caused aortic injury. A further one case was rated as grade II and REBOA was placed in the distal area to the aortic injury. This patient was discharged without the operation and was followed-up in the outpatient setting by the vascular surgery department. Mesenteric ischemia was clinically diagnosed based on bloody stool, elevated levels of lactic acid, creatinine kinase, lactate dehydrogenase, and elevated amylase despite the absence of pancreatic injury on the initial CT scan. Ischemia of a lower extremity was clinically diagnosed based on pulseless, pallor, and coldness at extremities of the side where REBOA was placed. Thrombus or occlusion of the mesenteric artery or artery in lower extremities were not confirmed by imaging modalities such as ultrasound or contrast-enhanced CT scan. The sheaths were placed for a median of 412 min (IQR 166–739) in 22 cases with available data in our study. In two patients with ischemic complications, the sheath was placed for 1054 min and 534 min, respectively. During this period, REBOA was intermittently inflamed and deflated because the patients were hemodynamically unstable and hypotensive. Patients who had these ischemic complications died of multiorgan failure in the intensive care units.

**Table 3:** Complications of REBOA

Complications	
Mesenteric ischemia (*n*, %)	1, 4.2%
Ischemia of lower extremities (*n*, %)	1, 4.2%
Insertion to patients with thoracic aorta injury (*n*, %)	3, 12.5%

*REBOA* resuscitative endovascular balloon occlusion of the aorta

## Discussion

This was a retrospective analysis of a clinical series of REBOA for the control and resuscitation of traumatic hemorrhage in four Japanese tertiary care hospitals. The first case series describing REBOA was published in 1954 [15]. In 1989, Gupta et al. published a case series of patients with penetrating abdominal trauma and reported that 33% of the patients survived until hospital discharge [16]. Brenner et al. described a case series of REBOA with an overall survival rate of 66% [17], whereas a case series by Irahara et al. reported a survival rate of 36% [18]. A report by Saito et al. indicated a 30-day survival rate of 29.2% [19], whereas an in-hospital survival rate of 32% following REBOA was reported by Moore et al. [20]. The in-hospital survival rate of 41.7% observed in the present case series is consistent with most of these earlier case series [16, 18–20]. Considering that well-trained trauma surgeons were involved in previous studies [19, 20], the present study suggests that emergency physicians can feasibly place REBOA and effectively control hemorrhage, thereby gaining time in emergency departments where trauma surgeons may not be readily available for 24 h a day, 7 days a week. The majority of the Japanese emergency rooms do not have trauma surgeons, and general surgeons are called instead. However, given that trauma surgery is a highly specialized subject, deciding whether to perform surgery for damage control or interventional radiology is not always easy for a general surgeon. In the present study, REBOA was performed by emergency physicians in 22 of 24 cases, and by general surgeons in 2 of 24 cases. This finding illustrates REBOA as a potentially useful tool that can be utilized by emergency physicians to gain time in the management of hemorrhagic shock in emergency rooms that do not have a trauma surgery department.

In a systematic review, Morrison et al. reported that the overall rate of morbidity with REBOA was 3.7% [9]. However, in the present study, five patients (20.8%) had complications associated with REBOA including placement of REBOA in thoracic aortic injury in three patients (12.5%), limb ischemia in one patient (4.2%), and mesenteric ischemia in one patient (4.2%). Since 50.0% of the patients did not survive beyond 24 h, other potential latent complications of REBOA cannot be ruled out.

Although Taylor et al. concluded that REBOA could be implemented safely without ischemic extremity complications [21], the majority of the reports describing ischemic complications are from Japan [19, 22]. A recent international study revealed that 7% of the patients undergoing REBOA had ischemic complications such as extremity compartment syndrome [23]. These adverse events were reported only in the continuous occlusion group in whom REBOA was fully inflated during the entire procedure. It should be emphasized that REBOA is a strategy that should be reserved for temporary management of hemorrhagic shock and should be discontinued as soon as possible to prevent ischemic complications.

It is reported that the use of REBOA in the setting of thoracic hemorrhage can be potentially risky since it could exacerbate the bleeding from the great thoracic vessels [24]. However, in the present case series, the REBOA was placed routinely in zone I as it might have been difficult to place REBOA adequately to cover only the affected blood vessels since we could not use fluoroscopy in the emergency rooms. Recently, the use of ultrasound has been suggested to confirm the position of the balloon [25]. This technique may make it possible to place the balloon in a more accurate place in the emergency room without fluoroscopy.

Opportunities to use REBOA are limited in Japan where traumatic injury is uncommon. The sufficient number of cases to use REBOA for the safe placement of REBOA is unclear. Among the four hospitals included in this study, REBOA was performed in an average of 5.6 cases per year or 24 cases per 51 months. Other Japanese studies reported similar rates ranging from 3.3 to 4 cases per year [18, 19]. Conversely, REBOA was performed in 8.1–18 cases per year as reported in studies from the United States [5, 17, 20].

Another unanswered question is who should place REBOA. Emergency physicians, trauma surgeons, and interventional radiologists can be involved in the immediate and direct care of trauma patients. Emergency physicians control airway, respiration, and hemodynamics to gain time for definitive therapy, whereas trauma surgeons and interventional radiologists definitively stop bleeding in elective settings. Emergency physicians and interventional radiologists routinely place sheaths, a necessary technique in REBOA. However, opportunities to become ready for safe REBOA implementation are limited for any physician. In the present study, emergency physicians performed REBOA in most cases (22/24), and four emergency departments included in this study are standard Japanese emergency departments that are not specialized in trauma care. Conversely, most of the studies from other countries reported that REBOA was performed at level 1 trauma centers by trauma surgeons who completed an educational course for REBOA or who were experienced in vascular surgery [5, 17, 20, 26]. Therefore, emergency physicians should exercise caution regarding the complications of REBOA in situations with no or little support from trauma surgeons. However, REBOA can be a useful approach to gain time during the management of hemorrhagic shock.

There is currently no consensus on the correct approach for the placement of the catheter and confirmation of the appropriate tip placement during REBOA. Trauma surgeons are not always available, and transferring patients to the operation room or the interventional radiology laboratory can be risky in hemodynamically unstable patients. Fluoroscopy is not available in the emergency department in most Japanese hospitals, and physicians are required to place REBOA in the emergency room. In all the cases in the present study, REBOA was inserted without fluoroscopy in the emergency rooms. There were three patients complicated with thoracic aortic injury for whom REBOA was placed. In their systematic review, Morrison et al. reported that fluoroscopy was used to aid and confirm REBOA balloon deployment and placement in 64.4% of the reported cases [9]. Conversely, a recent Japanese study described that REBOA was placed without imaging in 92% of patients and that imaging was used to confirm the guidewire location in 77% of the patients (15%, 29%, and 32% using ultrasound, plain x-ray, and fluoroscopy, respectively). Ogura et al. reported the usefulness of ultrasound-guided REBOA [25]. Furthermore, Moore et al. described a protocol for REBOA implementation that utilized routine chest x-ray to determine whether aortic injury occurred as a complication [5, 20]. In the present case series, aortic injuries were not identifiable by chest x-ray, and the combined use of chest x-ray and abdominal ultrasound to screen for aortic injury is a reasonable approach, as these tests are quick and most emergency departments have this equipment.

As discussed before, variations in REBOA practices across countries might have led to the observed difference in patient outcomes in the current study.

Additionally, some studies suggested that smaller introducer sheaths for REBOA might be associated with fewer complications [3, 27]. In the present study, 7-French smaller introducers were used in two cases that survived, without any complications. Matsumura et al. suggested that partial occlusion of the aorta might allow for extension of the occlusion duration without a reduction in survival rate [27]. Smaller introducers and partial occlusion of the aorta might be a safer and more effective approach for emergency physician-led REBOA in an emergency room setting; however, replication of the results of the current study is warranted.

In the present case series, none of our patients had apparent reperfusion injury. A previous animal study described that calcium administration and insulin and glucose therapy were required for hyperkalemia secondary to reperfusion injury after prolonged REBOA use [28]. Therefore, we need to be cautious about reperfusion injury and blood tests, especially the serum potassium level, and arterial blood gas analysis should be performed frequently to find early evidence of reperfusion injury.

There are no clear criteria regarding the optimal timing of termination of the balloon occlusion. The current guideline recommends slow balloon deflation over a period of 5 min [29] since blood pressure can suddenly drop due to the much larger blood volume distribution after the deflation, even if the hemorrhage itself is well-controlled.

## Limitations

The present case series has several limitations. The number of patients was not sufficient to evaluate the feasibility and safety of REBOA. As a retrospective study, the present study did not include clear criteria for REBOA. However, systolic blood pressure was less than 90 mmHg in almost all patients who underwent REBOA in the current study. Thus, REBOA may be useful for controlling massive hemorrhagic shock in ultimately critical situations. Large-scale prospective studies are warranted to confirm the feasibility and safety of REBOA.

## Conclusion

REBOA can be an effective and feasible tool to control massive hemorrhage due to trauma. However, caution should be exercised regarding complications including placement of REBOA in aortic injury and limb ischemia in cases where REBOA is performed in an emergency department setting with minimal or no support from trauma surgeons.

## Abbreviations

CT: Computed tomography
IQR: Interquartile range
ISS: Injury Severity Score
REBOA: Resuscitative endovascular balloon occlusion of the aorta
RTS: Revised Trauma Score
TRISS: Trauma and Injury Severity Score
